# Antitumor Applications of Photothermal Agents and Photothermal Synergistic Therapies

**DOI:** 10.3390/ijms23147909

**Published:** 2022-07-18

**Authors:** Chaowei Li, Yue Cheng, Dawei Li, Qi An, Wei Zhang, Yu Zhang, Yijun Fu

**Affiliations:** 1 School of Textile and Clothing, Nantong University, Nantong 226019, China; 13063589580@163.com (C.L.); cy970218@126.com (Y.C.); anqi960110@163.com (Q.A.); zhangwei@ntu.edu.cn (W.Z.); z.yu@ntu.edu.cn (Y.Z.); 2 National & Local Joint Engineering Research Center of Technical Fiber Composites for Safety and Health, Nantong University, Nantong 226019, China

**Keywords:** photothermal therapy, photothermal transduction agents, nanoparticles, antitumor

## Abstract

As a new tumor treatment strategy, photothermal therapy (PTT) has the advantages of accuracy, ease of administration, a high efficiency and low side effects. Photothermal transduction agents (PTAs) are the key factor which play an important role in PTT. The mechanism of PTT is discussed in detail. The photothermal conversion efficiency (PCE) can be improved by increasing the light absorption and reducing the light scattering of photothermal conversion agents. Additionally, non-radiative relaxation path attenuation can also promote energy conversion to obtain a higher value in terms of PCE. The structure and photothermal characteristics of various kinds of PTAs (metal materials, carbon-based nanomaterials, two-dimensional nanomaterials, and organic materials) were compared and analyzed. This paper reviews the antitumor applications of photothermal synergistic therapies, including PTT combined with immunotherapy, chemotherapy, and photodynamic therapy. This review proposes that these PTAs promote the development of photothermal synergistic therapies and have a great potential in the application of tumor treatment.

## 1. Introduction

According to a recent World Health Organization report on cancer, one out of every six people dies from the disease. Surgery, chemotherapy, radiation, immunotherapy, targeted therapy, and other treatments are currently used to treat cancer [1,2]. Surgical treatment has played an essential role in removing malignancies from the body since the invention of anesthesia in 1846, especially when paired with chemotherapy and imaging technology. Surgical treatment, however, is still ineffective due to the difficulty of removing microscopic tumor cells that are simple to disseminate and promote tumor recurrence [3,4]. Chemotherapy is frequently used in conjunction with one or more than one other medication to kill cancer cells [5], and the drugs can circulate to most tissues and organs throughout the body for systemic treatment [6]. However, chemotherapy has some defects, for example, the growth of drug resistance, the nonspecific systemic dispersion of antineoplastic agents, low concentrations of medications reaching the target, and large toxic side effects, which have all contributed to major treatment consequences, such as hindering the proliferation of normal cells and damaging human tissues and organs [7,8].

Radiation therapy is one of the main modalities of clinical oncology, which destroys cancer cells by generating localized ionizing radiation to achieve anti-tumor effects [9]. This treatment is critical for patients whose tumors are inoperable or incompletely removed as well as those with recurrent disease [10]. In some cases, radiotherapy can treat tumors by inducing immunogenic cell death to induce systemic immunotherapy effects [11,12], and local irradiation has also been reported to eradicate treated tumors [9]. Unfortunately, radiation therapy can cause radiation damage to adjacent normal tissue, resulting in scar tissue that leads to systemic immunosuppression. In addition, the inherent resistance of tumors to radiotherapy, the lack of oxygen in the tumor microenvironment, and strong systemic and local toxic side effects greatly limit the efficacy of cancer radiotherapy [13,14]. As a result, novel tumor therapy strategies with less damage to normal cells and better therapeutic impacts are required.

Immunotherapy, gene therapy, photodynamic therapy (PDT), and photothermal therapy (PTT) are examples of new cancer treatments that have considerably enhanced the effectiveness of tumor treatment [15,16,17,18,19]. Due to its advantages of a low cost, high efficiency, minimal invasiveness, few side effects, and strong targeting, photothermal treatment has garnered a large amount of interest in tumor therapy [20,21,22,23]. Photothermal transduction agents (PTAs) are used in PTT to convert light energy into heat energy. PTAs are injected into the body, and, using targeting technology, they accumulate near tumor tissue, where they damage the protein structure of tumor cells, resulting in a cure. PTT has the advantage of being able to use variable levels of external laser irradiation to precisely target the tumor while avoiding harm to the surrounding healthy tissue; it can also be used to treat all types of cancer and is non-invasive [24,25,26].

The major issue with PTT at this juncture is its low depth of light penetration, which can result in the inadequate ablation of malignancies outside of the irradiation area. Furthermore, the delivery PTAs in tumors is inefficient, the overheating of tumor areas leads to unnecessary damage to normal tissues, and there is chance of the development of drug resistance.

The mechanism of PTT was investigated in this work, and many forms of PTAs were introduced. The use of PTT in combination with immunotherapy, chemotherapy, and PDT in tumor treatment was investigated, and the prospects of applying PTT are presented.

## 2. Mechanism of PTT

Studies have shown that the biochemical reaction rate of cells increases significantly with an increase in temperature, which is due to the increase in reactive oxygen species, the density in cells, oxidative stress, and oxidative damage to nucleic acids, lipids, and proteins [27]. In the near infrared (NIR) window (650–1000 nm), low light absorption and the transparency of healthy tissues allow for deep penetration into the body. PTT uses the strong light absorption and high photothermal conversion efficiency (PCE) of PTAs to absorb light energy and effectively convert it into heat energy, so as to increase the temperature of local tumors, which leads to the cell membrane rupture or protein degeneration of tumor cells, and avoid damage to normal cells [28,29,30]. Studies have found that factors such as tumor type, NIR light irradiation conditions, and the heat absorbed by cells all have a specific impact on PTT-induced cell death [31]. The main mechanism behind PTT involves a local high temperature inducing various changes in tumor cells. For example, PTT-induced hyperthermia triggers the release of antigens, pro-inflammatory cytokines, and immunogenic intracellular substrates from dying tumor cells, thereby promoting the activation of antitumor immunity [32]. Furthermore, PTT generates excess reactive oxygen species (ROS) under NIR irradiation, including hydroxyl radicals, hydrogen peroxide superoxide, and singlet oxygen [33]. Excessive ROS leads to the destruction of DNA, proteins, and lipids, eventually causing the apoptosis of cancer cells, which is considered to be another anticancer mechanism of PTT [34]. Prasad et al. reported that carbon dots can convert NIR light into heat and generate ROS, leading to 4T1 cell death and breast tumor ablation [35].

When irradiated by NIR light, PTAs can both absorb and scatter light. The sum of the two is called extinction. Only the absorbed energy can be partially converted into heat, and the absorption, scattering, and extinction coefficients of PTAs are related to the size, shape, and composition of PTA nanoparticles [25,36]. When photons radiated by NIR collide with small molecule chromophores, electrons are excited from their ground state (S_0_) to a higher singlet state (S_1_). They are then relaxed to the lowest excited state through internal conversion [37].

**Figure 1:**
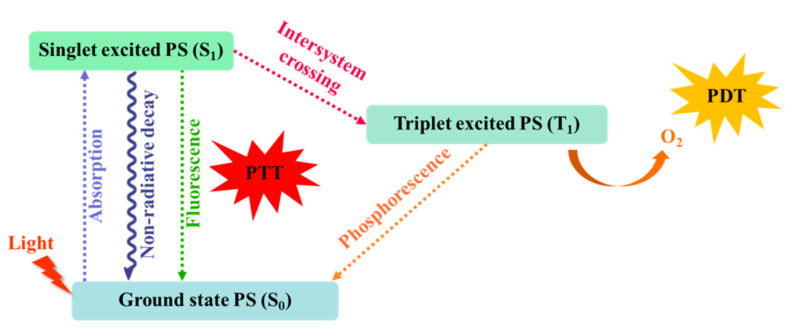
The Jablonski diagram of different energy transfer mechanisms.

The relaxed molecules at the lowest vibrational level of the excited state can undergo one of the following three processes: (1) they can return to the ground state by emitting photons (fluorescence); (2) they can decay back to the ground state (heat generation) following the non-radiative relaxation path [38]; and (3) they can transition from the singlet state to the triplet state (T_1_) through intersystem crossover (phosphorescence), as shown in Figure 1. The photothermal effects are usually the result of the attenuation of non-radiative relaxation paths [37,39,40]. Therefore, the enhancement of PCE can be achieved by increasing the sensitivity of PTAs to light, reducing light scattering, and promoting energy conversion through non radiative relaxation path attenuation.

## 3. Photothermal Transduction Agents

PTAs facilitate light absorption and light conversion to heat. The ideal photothermal conversion agent should have good photostability, an NIR absorption capacity, a high PCE, and good biocompatibility [41,42].

PTAs include inorganic materials and organic materials. Inorganic materials include noble metals (e.g., gold nanoparticles and platinum nanoparticles) [43,44], carbon-based nanomaterials (e.g., carbon nanotubes, graphene, graphene oxide, carbon quantum dots, and mesoporous carbon) [40,45,46,47]. Organic materials include NIR-responsive small molecules and semiconducting polymer nanoparticles [48,49,50] (e.g., cyanine, porphyrin, copper phthalocyanine, and diketone pyrrole pyrrole).

Generally speaking, inorganic PTAs have higher PCE and better photothermal stability than organic PTAs, but organic PTAs have greater biodegradability and biocompatibility compared to inorganic PTAs [25,51]. With a large number of studies on spherical and rod-like morphologies, the exploration of two-dimensional (2D) nanomaterials in the fields of sensing [52,53], catalysis [54], device manufacturing [55], and energy storage [56,57] has increased greatly in recent years [58]. Common 2D materials include black phosphorus, nanosheets, boron nitride, and graphitic carbon nitride [59], etc. Scientists are trying to overcome the shortcomings of different types of materials so as to further improve the effect of tumor treatment.

### 3.1. Metal Materials

Metal materials with high oxidation resistance are the most studied inorganic PTAs, with these including Au, Ag, Pt, and Pd [60]. They can absorb laser to excite electrons from the ground state to the excited state, and they can then release energy in the form of heat through nonradiative decay. Because precious metal materials have the advantages of easy surface modification and good biological stability, these factors can improve the PCE [61]. Currently, the gold nanostructures studied include gold nanorods [62], gold nanoshells [63], gold cages, gold nanorings [64], gold nanoparticles [65], rice vesicles, and chiral gold nanoparticles [66]. Since PTT containing gold nanoparticles was first reported in 2003, the research on gold nanomaterials has attracted extensive attention [67].

Nishikawa et al. [68] prepared an injectable anti-shear hydrogel composed of gold nanorods and nanospheres linked by CpG oligonucleotides. NIR irradiation hydrogel can stimulate heat shock protein 70 and mRNA expression, while reducing the volume of primary tumors. Gold nanomaterials have also been proved to have strong photothermal efficiency and can be used in combination with immunotherapy. In addition, Liu et al. [69] proposed gold-shell silica nanoparticles, and Mohammad et al. [70] proposed gold-shell Fe_3_O_4_ nanoparticles. Due to the hollow nanostructure of gold nanoshells, these materials show high light absorption in the NIR band.

Although metal materials provide an excellent photothermal effect for cancer treatment, there remain a number of problems which need to be solved: (1) gold nanomaterials are not biodegradable, so it is necessary to further detect the treated gold nanomaterials, and (2) gold nanomaterials have poor optical stability. Under continuous NIR irradiation, some specific gold nanomaterials are deformed due to the “melting effect” [71], and the photothermal effect is reduced. Therefore, precious metal materials need to be studied further.

### 3.2. Carbon Based Nanomaterials

Carbon based nanomaterials, including carbon nanotubes and graphene, have attracted much attention due to their excellent photothermal properties and biocompatibility in the medical field [72]. Among them, carbon nanotubes can effectively combine chemotherapy with PTT to enhance the anti-tumor effect. As carbon nanotubes have high a specific surface area and high PCE, they can be used as potential carriers for the transport of nucleic acids, proteins, and drug molecules in cells. They are therefore being employed in the development of the next generation of PTAs [73,74].

In order to further improve the PCE, multi-walled carbon nanotubes (MWCNT) have been developed. These consist of a nested columnar structure with diameters ranging from several nanometers to several microns. Compared with single-walled carbon nanotubes (SWNT), they can load more drugs and absorb more NIR radiation, making them an ideal medium for chemical–thermal combination therapy [75,76,77].

**Figure 2:**
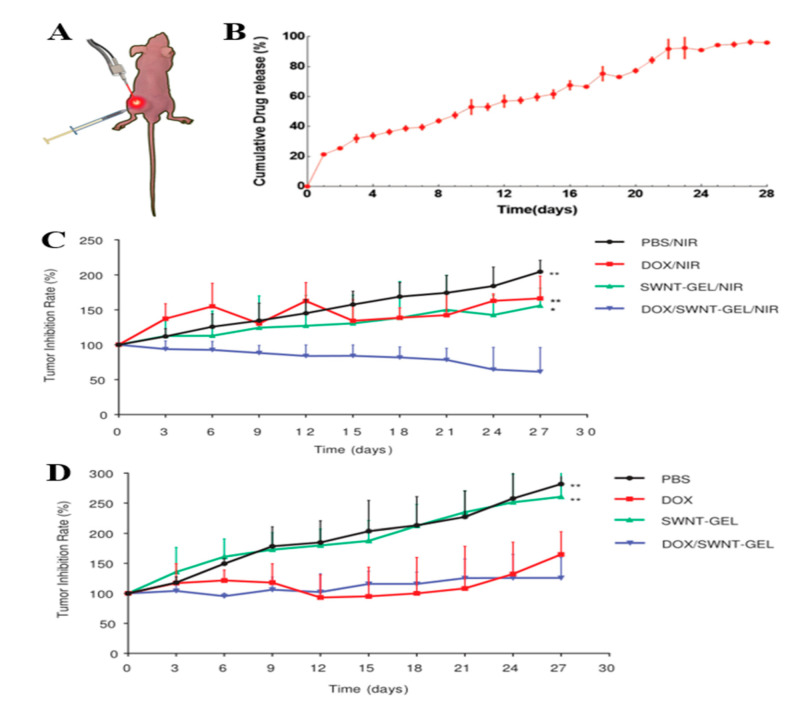
(**A**) The xenograft tumor model was established by subcutaneous injection of BGC-823 gastric cancer cells in mice. DOX/SWNT-GEL was injected into the tumor, and then the mice received NIR laser irradiation at the tumor site; (**B**) DOX release curve of SWNT-GEL in PBS for 28 days at a constant temperature of 43 °C; (**C**) tumor growth rate of mice treated with different methods and NIR radiation; (**D**) tumor growth rate of mice with different treatments without NIR radiation. Data was presented as mean ± SD (* indicates *p* < 0.05 and ** denotes *p* ≤ 0.01, compared with DOX/SWNT-GEL/NIR or DOX/SWNT-GEL group). Reproduced with permission from ref. [78] Copyright (2015). Wiley-VCH Verlag.

Zhou et al. [78] developed SWNT-based thermosensitive hydrogels for the treatment of xenograft gastric cancer mice, as shown in Figure 2. First, injectable hydrogels can deliver the anticancer drug doxorubicin hydrochloride (DOX) directly to the tumor site, enabling it to play an anticancer role in situ. At the same time, NIR light can provide hyperthermia by penetrating tissue to stimulate SWNT located at the tumor site. The DOX released by the drug loading system exceeded 20% on the first day of injection, and the cumulative release reached approximately 96% on the 28th day, indicating that SWNT-GEL had the function of sustained drug release. In addition, after the intratumoral injection of SWNT, the tumor growth rate of mice was 156% with NIR and 261% without NIR after 28 days of treatment, respectively. The disease of the mice continued to be in remission under NIR irradiation.

### 3.3. Two-Dimensional Nanomaterials

2D nanomaterials have the advantages of an ultra-thin atomic thickness, a low toxicity, a controllable size, and a large specific surface area. They have unique physical and chemical properties, an easy surface modification, a high photothermal efficiency, and can promote the extension of tumor blood circulation and improve the accumulation capacity. 2D nanomaterials mainly include graphene and its derivatives, metal compounds [79,80], transition metal dihydroxy compounds (TMDC) [81], black phosphorus (BP) [82,83], and transition metal carbides/carbonitrides (MXene) (e.g., carbides, nitrides, or carbonitrides) [84,85,86].

#### 3.3.1. Transition Metal Dihydroxy Compounds

TMDCs include MoS_2_, WS_2_, TiS_2_, and MoSe_2_, which have high PCE, a good biocompatibility, and photothermal stability [87]. A TMDC is basically a layer of transition metal atoms (such as Ti, Zr, Hf, V, Nb, Ta, Mo, and W) sandwiched between two layers of sulfur atoms (such as S, Se, and Te). In particular, molybdenum disulfide (MoS_2_) shows good absorption characteristics in the NIR spectrum range, the element Mo widely exists in the identifiable elements of some enzymes, and the content of S in the body is rich, so MoS_2_ is biocompatible. In 2013, it was first proved that MoS_2_ nanoparticles were a new PTT material similar to graphene oxide (GO). Compared with gold nanorods, they have greater capacity for NIR absorption. However, MoS_2_ and higher PCE (62.5%) materials disperse poorly in aqueous solution and cannot locate specific tissue sites, which limits their application in targeting cancer nanodrugs. Therefore, MoS_2_ is often used in PTT and other combination therapies [88].

**Figure 3:**
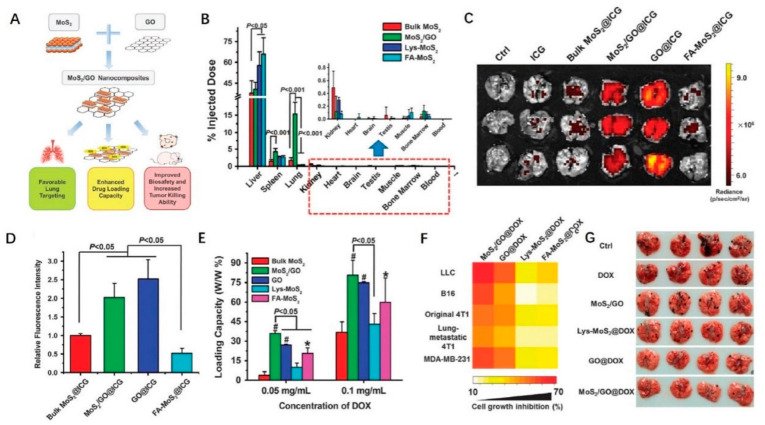
MoS_2_/GO nanocomposites. (**A**) The overall schematic diagram depicting functionality and biocompatibility enhancement through the synthesizing of MoS_2_/GO nanocomposites; (**B**) in vivo biodistribution of various materials in mice. The Mo contents were examined in mice 24 h post i.v. injection. The results from ICP-MS determination were shown as % of injected. In vivo imaging and lung accumulation analysis; (**C**) ICG fluorescent images of lungs from mice 24 h post-injection of free ICG and ICG-loaded nanomaterials; (**D**) quantification of relative ICG fluorescence in lungs (*n* = 3). ICG, indocyanine green. DOX loading capacity and tumor killing efficacy of different materials; (**E**) DOX loading capacity of nanomaterials (*n* = 4). * indicates *p* < 0.05 and # denotes *p* < 0.001, compared to bulk MoS_2_-treated group; (**F**) in vitro tumor killing efficacy of DOX-loaded materials at the same mass concentrations (*n* = 5). The concentrations of materials were tailored for each type of cells as follows: 2 μg·mL^−^^1^ for LLC cells, 6 μg·mL^−^^1^ for B16 cells, 30 μg·mL^−^^1^ for 4T1 cells, and 15 μg·mL^−^^1^ for MDA-MB-231 cells; (**G**) representative images of metastatic tumor nodules in the lungs from treated and untreated mice with implantation of B16 murine melanoma cancer cells. DOX, doxorubicin; LLC, Lewis lung carcinoma. Reproduced with permission from ref. [89]. Copyright (2018). Springer Nature.

Because GO is highly soluble in aqueous solution, it exhibits obvious targeting, a high absorption, and an excellent PCE in the NIR range. Liu et al. [89] doped GO monolayers onto an MoS_2_ layer to prepare MoS_2_/GO nanocomposites. Figure 3 shows the schematic diagram of its structure and therapeutic mechanism. The PCE was as high as 42% when photoacoustic imaging and PTT were performed simultaneously in live mice. Tissue targeting studies have shown that MoS_2_/GO composite material preferentially accumulates in the lung, indicating that MoS_2_/GO composite material targets the lungs, indicating a direction for the treatment of emphysema diseases such as chronic bronchitis and bronchial asthma.

#### 3.3.2. Transition Metal Carbides/Carbonitrides

MXene has an energy band structure similar to that of semi-metal, that is, a local surface plasmon resonance effect similar to that of metal nanoparticles [90,91]. It exhibits an excellent electromagnetic wave absorption performance and dissipates the absorbed wave in the form of heat inside the substance. It is a new 2D material with a wide range od applications. MXenes used for photothermal effect include titanium carbide, niobium carbide, and tantalum carbide [92,93] The most commonly used are titanium carbide nanoparticles.

Li et al. [94] used a Ti_3_AlC_2_ ternary-layered carbonitride (MAX) phase as a raw material and etched an Al layer with hydrofluoric acid (HF) aqueous solution. Under the action of ultrasound, the pretreated HF-treated MAX phase was immersed in DMSO to form a peeling single layer or a small amount of layered MXene flakes. The absorption peak of MXene is about 800 nm, while CNT absorbs a wide spectrum of 300–1300 nm, but there is no obvious absorption peak, so it can be seen that MXene has a higher light absorption capacity than CNT. In addition, no matter if the wavelength of the laser source is 473 nm or 785 nm, the PCE of MXene is close to 100%, which indicates that MXene is an excellent photothermal material.

#### 3.3.3. Black Phosphorus

Since 2015, BP nanomaterials have attracted significant attention in the biomedical field because of their high PCEs, large specific surface areas, good biocompatibility, and biodegradability. As metal-free layered semiconductor materials, BP nanomaterials have a thick-dependent band gap and can be tuned from 0.3 eV in volume to 2.0 eV as a single layer. BP usually exists in the form of nanoparticles, quantum dots, and nanosheets [95]. Compared with 2D materials such as graphene and MoS_2_, BP has a higher surface volume ratio due to its folded lattice structure, which can improve drug loading. BP is also photostable, biocompatible, biodegradable [96], and can be easily combined with other cancer treatments due to its absorbance across the entire NIR range.

**Figure 4:**
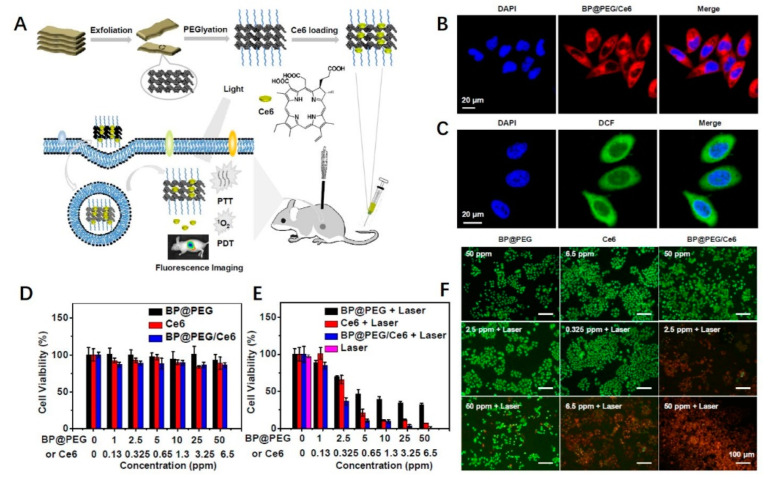
(**A**) Schematic representation of BP@PEG/Ce6 NSs preparation and its application as fluorescence and thermal imaging guided photothermal and photodynamic cancer therapy; (**B**) fluorescence images of HeLa cells cultured with BP@PEG/Ce6 NSs; (**C**) intracellular ROS generation of HeLa cells treated with BP@PEG/Ce6 NSs and irradiated with 660 nm laser. Relative viabilities of HeLa cells after being treated with BP@PEG NSs, Ce6, and BP@PEG/Ce6 NSs at different concentrations of BP@PEG NSs (1, 2.5, 5, 10, 25, and 50 ppm) or Ce6 (0.13, 0.325, 0.65, 1.3, 3.25, and 6.5 ppm); (**D**) without and (**E**) with irradiation (660 nm, 0.65 W·cm^−2^, 10 min); (**F**) fluorescence images of HeLa cells co-stained with Calcein AM (live cells, green) and PI (dead cells, red) upon the addition of BP@PEG NSs, Ce6, and BP@PEG/Ce6 NSs without and with irradiation (660 nm, 0.65 W·cm^−^^2^, 10 min). Reproduced with permission from ref. [97]. Copyright (2018). American Chemical Society.

Yang et al. [97] modified BP nanosheets with polyethylene glycol (PEG) and loaded with chlorin (Ce6) photosensitizer to obtain BP@PEG/Ce6 NSs (as shown in Figure 4). After injection into mice, BP@PEG/Ce6 NSs was transmitted to cervical cancer cells through an enhanced permeability and retention effect, showing good tumor targeting. In vitro experiments have shown that BP@PEG/Ce6 NSs can effectively produce photoheat and reactive oxygen species by releasing Ce6 photosensitizers, thus increasing cell membrane permeability and drug uptake. The related PCE is 43.6% under the NIR light at 660 nm. This material has potentially broad applications in the treatment of PTT.

In addition, some metal compounds with biocompatibility, such as oxides, sulfides [98], and selenides, etc., can also be used as PTAs in cancer therapy due to their plasmon resonance properties [99,100].

### 3.4. Organic Materials

So far, organic PTAs based on NIR response small molecules (anthocyanin, porphyrin [101], phthalocyanine [102], and theobromine [103]) and semiconductor polymer nanoparticles (polyaniline [104] and polypyrrole [105]) have shown excellent therapeutic efficacy and have been widely studied as potential PTAs.

PTAs based on small organic molecules, such as cyanine dyes and porphyrins, are often used in cancer imaging and treatment. However, they have poor water solubility, limited tumor accumulation, and low photobleaching and photothermal efficiency. In order to solve this problem, organic nanocarriers can be wrapped to improve solubility and pharmacokinetics, enhance tumor penetration and retention in vivo, and improve photobleaching and photothermal efficiency [5]. So far, organic nanocarriers such as polymer micelles, vesicles, and liposomes have been widely used in photothermal cancer treatment.

**Figure 5:**
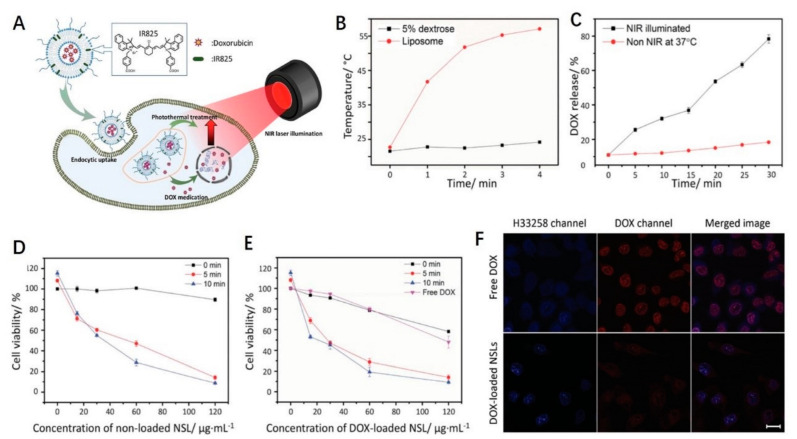
IR825 dye as a photothermal conversion agent. (**A**) Schematic illustration showing the structure and action mechanism of NIR-absorptive DOX-loaded stealth liposome; (**B**) heating curves of the hydration solution (5% dextrose) and the NSL solution upon continuous NIR laser illumination at a laser power density of 0.5 W·cm^−2^; (**C**) DOX release profiles of DOX-loaded NSL at physiological temperature and under NIR light irradiation (power density: 0.5 W·cm^−2^), respectively. Relative viabilities of HeLa cells incubated with (**D**) nonloaded NSL and (**E**) DOX-loaded NSL at various concentrations without and with NIR laser illumination for 5 and 10 min, respectively; (**F**) confocal laser fluorescence spectroscopy images of live HeLa cells incubated with free DOX (3 μg·mL^−^^1^) or DOX-loaded NSL (200 μg·mL^−^^1^) solutions. The cell nuclei were stained with H33258. Scale bar is 10 μm. Reproduced with permission from ref. [106] Copyright (2015). Wiley-VCH Verlag.

As a photothermal conversion agent, IR825 dye has a high absorption coefficient and low fluorescence quantum yield. However, due to its extremely low water solubility and minimal absorption efficiency, therapeutic applications are greatly limited. By using nanocarriers, Li et al. [106] incorporated IR825 into heat-responsive liposomes to enhance bioavailability and photothermal effects in vivo, as shown in Figure 5. The results show that the material retains the high PCE and high photothermal properties of IR825. In vitro and in vivo experiments confirmed that DOX-loaded IR825 mixed with thermoresponsive liposomes combined with PTT can achieve a better tumor suppressive effect than liposomes, DOX, or photothermal ablation alone.

## 4. Photothermal Synergistic Therapies

As PTT penetrates into tissues, the power of light may decrease, and the uneven heat distribution and severe hypoxia in tumor tissues in PTT make it difficult to eradicate tumors in a single mode of treatment. Therefore, collaborative therapy is an effective way to solve these problems [107]. First, synergistic therapy helps to reduce side effects because the dose of each therapy can be reduced while the overall therapeutic effect can be maintained. Second, interactions between different treatments can produce greater efficiency. For example, heat generated by the photothermal effect can improve the efficacy of some anticancer drugs [108,109].

NIR light (wavelength 650–900 nm) has the characteristics of weaker tissue absorption and larger tissue penetration depth, which is more suitable for PTT [32]. NIR-induced heat production can not only lead to the damage of malignant cells, but also enhance the efficacy of other treatment modalities, thus enabling photothermal synergistic therapy. Photothermal synergistic therapy can be achieved through different working mechanisms such as the photothermal-controlled release of therapeutic drugs, photothermal-enhanced enzyme activity, photothermal-regulated gene expression, photothermal-triggered immune response activation, and photothermal-enhanced chemical reactions [110].

### 4.1. PTT and Immunotherapy Synergistic Therapy

Immunotherapy is an effective method for cancer treatment. It destroys cancer cells by activating the human immune system on the basis of tumor immunology [111]. The combination of PTT and immunotherapy can achieve an synergistic antitumor effect. On the one hand, PTT-induced cancer cell death can release a tumor specific antigen in situ and trigger a specific anti-tumor immune response. On the other hand, patients can eliminate diffuse metastases beyond the scope of the laser irradiation range and prevent tumor recurrence by relying on their own immune function [112,113].

Wang et al. [114] reported that thermal ablation-induced immune responses based on SWNT could not fully inhibit the growth of secondary tumors. Anti-CTLA4 antibody was introduced after the thermal ablation of the primary tumor, which greatly improved the efficacy and directed the immune checkpoint pathway of abnormal immune response to PD-L1 and IDO.

Chen et al. [115] introduced the in situ autologous tumor vaccine for the combined treatment of PTT and immune adjuvant GC targeting primary tumors. The advantage of the vaccine is the ability to treat different tumors as antigens derived from the tumor itself, so that any subtle differences in the antigen spectrum are captured accordingly.

### 4.2. PTT and Chemotherapy Synergistic Therapy

While killing cancer cells, chemotherapy drugs may also cause collateral damage to normal cells through oxidative stress [116]. Due to the synergistic effect of PTT and chemotherapy, the ability of tumor metastasis can be controlled to achieve targeted therapy and reduce damage to normal tissues [117]. Currently, much research is focused on developing nanosystems for drug delivery.

**Figure 6:**
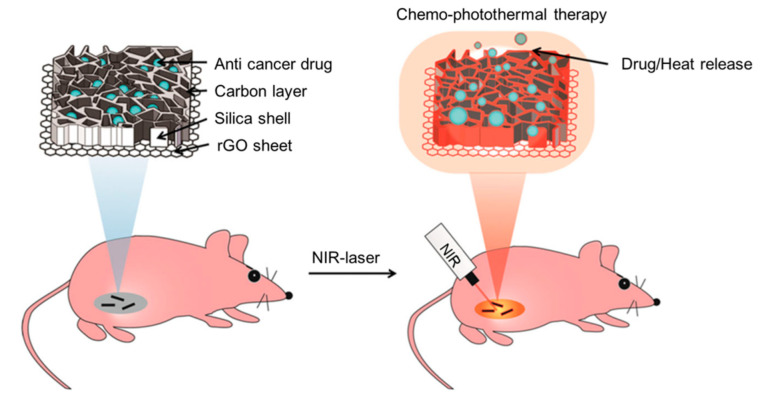
Schematic illustration of chemo-photothermal therapy using reduced graphene oxide/carbon/mesoporous silica nanocookies under NIR light-control. Reproduced with permission from ref. [118] Copyright (2014). Wiley-VCH Verlag.

Chen et al. [118] coated amorphous carbon on a mesoporous silica carrier on a rGO sheet and constructed a new photoresponsive drug carrier with a photothermal effect and nanometer biscuit structure, which can release drugs and generate a large amount of heat under NIR irradiation (as shown in Figure 6). With good biocompatibility and an efficient cell uptake, the material successfully cleared subcutaneous tumors within 14 days after 5 min of NIR irradiation without distal damage. It is an excellent new delivery platform for combined chemotherapy/hyperthermia.

Wang et al. [119] synthesized polyethylene imine-functionalized SWNT with the significant sustained release of doxorubicin. In order to achieve the chemo-photothermal therapy of tumors, polyethylenimine-modified SWNT were used as the drug carrier. Studies have shown that the DOX loading capacity (the mass ratio of DOX contained in the carrier to the carrier) reaches 248%, the slow release of DOX in animal tissues, and that the drug circulation time is significantly prolonged, which helps to reduce systemic toxicity. Therefore, these coupled treatment options offer prospective options for future research and development.

### 4.3. PTT and Photodynamic Synergistic Therapy

Compared with traditional therapies, PDT uses light source targeting to selectively eliminate primary and recurrent tumors, thus avoiding normal tissue damage, narrowing surgical scope, improving patient safety, activating immune function, and reducing recurrence [120]. PDT is made up of three basic components: light, PS, and oxygen. PDT involves mixing a photosensitizer into the target and then illuminating it with light at a wavelength corresponding to the photosensitizer’s absorption band [121]. After irradiation, type I and type II REDOX reactions will occur, leading to the generation of singlet oxygen and other superoxide ions. Finally, these singlet oxygen and superoxide ions can act as target killers [122,123]. PTT and PDT are two major non-invasive medical technologies in the treatment of cancer and other diseases [124]. The combined application of PTT and PDT can not only stimulate the efficiency of the photothermal enhancement of PDT, but also induce the synergistic effect, which can accelerate the flow of blood in the tumor and lead to more oxygen entering the tumor, thus enhancing the efficiency of PDT.

In order to compare the efficacy of porous PDT and PTT in hyperoxic and hypoxic tumors, Taratula et al. [125] studied, for the first time, the nanostructure-driven transformation of porphyrin PDT activation mechanism in an in vivo hypoxic tumor model. In addition, he also designed the application of boron platinate-dipyrrolidone and silicon-naphthalocyanine in phototherapy against tumors.

## 5. Conclusions and Outlooks

PTT is a novel approach to tumor treatment, and the advancement of PTAs in the production and use of PTT technology has aided its advancement. Taking advantage of the flexibility afforded in the design of nanomaterials, anticancer drugs, targeted carriers, or other therapeutic molecules could be incorporated into PTAs to achieve a combination of PTT, chemotherapy, PDT, immunotherapy, and other cancer treatment methods, thereby improving the therapeutic effect. Because the small penetration depth of light is the most significant barrier to PTT, the development relevant medical devices, such as ultra-micro fiber, is required, so that laser may be converted to deep tissue and play a therapeutic role. On the other hand, photothermal therapy agents must be delivered to tumor sites and the effects of PTT must be monitored using appropriate tools such as MRI and ultrasound.

## Data Availability

Not applicable.
